# Systematic Review: Is High-Energy Laser Therapy (HELT) With Flapless Corticotomy Effective in Accelerating Orthodontic Tooth Movement?

**DOI:** 10.7759/cureus.22337

**Published:** 2022-02-17

**Authors:** Rashad I. Shaadouh, Mohammad Y. Hajeer, Ghiath Mahmoud, Rashad M.T. Murad

**Affiliations:** 1 Department of Orthodontics, Damascus University Faculty of Dentistry, Damascus, SYR; 2 Department of Toxins and Pharmaceutics, Damascus University Faculty of Pharmacology, Damascus, SYR

**Keywords:** complications, treatment time, acceleration of tooth movement, upper incisors intrusion, canines retraction, laser-based flapless corticotomy, er: yag laser, high-energy laser therapy, lasers, accelerated tooth movement

## Abstract

The objective of this review was to critically and systematically appraise the available evidence regarding the effectiveness of high-energy laser therapy (HELT) with flapless corticotomy in accelerating orthodontic tooth movement and the associated untoward effects.

We searched eight databases electronically in August 2021: PubMed®, Medline®, Google Scholar, Cochrane Library, Scopus®, Web of Science™, Trip, and PQDT OPEN from ProQuest. Another search was done in the reference lists of the included studies. Randomized controlled trials (RCTs) were included in which patients had received fixed orthodontic treatment combined with HELT-assisted corticotomy in comparison with traditional orthodontic treatment. Cochrane’s risk of bias (RoB2) tool was used to assess the risk of bias.

Five RCTs and one CCT were included in this review (155 patients). The HELT-based corticotomy around the upper canines led to a greater canine retraction at the first and second months (P < 0.001). In the third month, no statistically significant differences were noticed. In one RCT focusing on incisor intrusion, the irradiated upper incisors showed a greater intrusion speed than that of the control group (4.587 mm in 59 days vs. 3.78 mm in 95.8 days, respectively). No significant side effects associated with the application of HELT were reported. According to the GRADE (Grading of Recommendations, Assessment, Development, and Evaluations) approach, the quality of evidence supporting these findings was low to moderate.

Although the acceleration of tooth movement appeared to be significant at least in the first two months, there was low to moderate evidence concerning the efficacy of HELT-based flapless corticotomy in the acceleration of orthodontic tooth movement. There is a need for more well-conducted high-quality RCTs.

## Introduction and background

Traditional orthodontic treatment for moderate to severe malocclusions usually takes several years when fixed appliances are used [1], and this can reduce patient compliance and cause multiple side effects [2]. A lot of subjects, particularly adults, refuse orthodontic treatment since it takes extended treatment time, especially if fixed appliances are used, which may have a negative impact on their daily life [3]. Thus, accelerating orthodontic treatment is a primary concern for both patients and clinicians.

In an attempt to accelerate orthodontic tooth movement, several approaches have been studied, including surgical approaches, such as corticotomy [4], distraction osteogenesis [5], and corticision and piezocision [6], and non-surgical approaches such as low-intensity laser irradiation, resonance vibration, pulsed electromagnetic fields, electrical currents, and biological approaches [7].

Dental lasers are generally divided into high-energy laser and low-level laser devices [8]. High-energy laser treatment (HELT) has a power output greater than 500 mW and can be used for cutting soft and hard tissues. Its energy ranges from hundreds to thousands of watts per square centimeter [8] such as neodymium-doped yttrium aluminum garnet (Nd: YAG) laser, argon laser, carbon dioxide (CO2) laser, erbium-doped yttrium aluminum garnet (Er: YAG) lasers, and the erbium-chromium yttrium scandium gallium garnet (Er, Cr: YSGG) lasers. High-energy laser therapy (HELT) is widely used in dentistry; it is used in caries removal, bacterial reduction during endodontic therapy, treatment of dentinal hypersensitivity, periodontal therapy, gingivectomy, and bone and soft tissue ablation [9]. In orthodontics, HELT has been shown to be effective in preventing enamel demineralization [10], preparation of surfaces for bracket bonding [11], debonding of ceramic brackets [12], and accelerating tooth movements [13].

Recently, HELT has been suggested to be used in performing alveolar corticotomy to induce the so-called 'regional acceleration phenomenon' (RAP) [13-14]. Erbium lasers can be used to perform flapless corticotomy due to their ability to ablate soft and hard tissues with minimal damage [15]. Therefore, they offer an alternative drilling modality to piezocision and conventional bur to drill holes, cut bone with minimal thermal damage, and have precise control of bone-cutting [16]. This causes transient demineralization and increases the activity of cells that accelerate tooth movement [17]. Therefore, it is considered one of the minimally invasive surgical methods of acceleration [13], whereas the low-level laser therapy (LLLT), which is considered one of the physical methods of acceleration, has been shown to affect bone remodeling by stimulating osteoclast, osteoblast, and fibroblast propagation, thereby accelerating orthodontic tooth movement [18].

Despite the importance of high-energy laser therapy and its widespread use in medical centers and teaching hospitals, few clinical trials have evaluated its efficacy as an adjunctive procedure for conducting corticotomies for accelerating tooth movement [13]. There are more than 10 systematic reviews and traditional reviews about the effectiveness of the LLLT in accelerating tooth movement [19-20], whereas, surprisingly, there is no single systematic review evaluating the effectiveness of high-energy laser therapy with flapless corticotomy in accelerating orthodontic tooth movement. Thus, the purpose of this systematic review was to critically and systematically appraise the existing evidence concerning the effectiveness of high-energy laser therapy in accelerating orthodontic tooth movement and the associated untoward effects with this procedure.

## Review

Materials and methods

A scoping PubMed search was performed to confirm the existence of similar systematic reviews and to investigate potentially relevant papers prior to writing the final systematic review protocol. No systematic review was found in the literature evaluating the effectiveness of high-energy laser therapy with flapless corticotomy in accelerating orthodontic tooth movement.

Eligibility Criteria

Exclusion and inclusion criteria were established according to the PICOS (Participants, Interventions, Comparisons, Outcomes, and Study design) framework:

Study design: Randomized controlled trials (RCTs) of any design: parallel-group design, split-mouth design, or compound design (two or more parallel-group designs with a split-mouth design in each group) and non-randomized controlled trials (CCTs) were included, without time-of-publication or language restrictions.

Participants: Healthy patients of all ages and malocclusions, both males and females, of all ethnic groups who received orthodontic treatment using a fixed orthodontic appliance were included.

Type of interventions: All types of treatment using fixed orthodontic appliances (with or without extraction) assisted by HELT-based corticotomies for accelerating orthodontic tooth movement were included.

Comparisons: Patients receiving traditional orthodontic treatment (without additional procedure to accelerate tooth movement) using fixed orthodontic appliances (with or without extraction).

Outcomes: The primary outcome was the rate of orthodontic tooth movement (RTM) or any measure indicating the effectiveness of high-energy lasers in accelerating teeth movements (i.e. treatment time, retraction time, the net of tooth movement, etc.). The secondary outcomes were patient-reported outcomes (pain, discomfort, alteration in mastication, other experiences, and satisfaction), loss of anchorage, and unwanted tooth movements. In addition, the other secondary outcomes were gingival and periodontal problems (gingival recession, loss of attachment, depth of probing, bone resorption), iatrogenic harm to teeth (vitality loss, root resorption), or stability of treatment in the long term.

Search Strategy

The following databases were electronically searched in August 2021: PubMed®, Medline®, Google Scholar, Cochrane Library databases, Scopus®, Web of Science™, Trip, and PQDT OPEN (to identify dissertations and theses).

The following terms and their derivatives were used: (Orthodontic) AND (Acceleration) AND (high-energy laser). Table 1 shows the details of the electronic search strategy. Another search in the reference lists of the included studies was done for any possible related paper that may have not been discovered by the electronic search.

**Table 1:** Electronic search strategy

Database	Search Strategy
CENTRAL (The Cochrane Library)	#1 orthodontic* OR "Tooth movement" OR "orthodontic tooth movement" OR "Tooth displacement " OR "orthodontic Treatment" OR "orthodontic Therapy" #2 accelerat* OR rapid* OR short* OR speed* OR fast OR velocity OR duration OR rate OR time OR "regional accelerated phenomenon" OR RAP. #3 laser OR high intensity laser therapy OR HELT OR hard laser OR high-energy laser OR Erbium lasers OR Er: YAG laser OR Er,Cr:YSGG laser OR CO2 laser OR Nd:YAG laser. #4 laser-assisted corticotomy OR lasersicion OR laser-assisted* OR laser induced* OR laser decortication. #5 #3 OR #4 #6 #1 AND #2 AND #5
PubMed	#1 orthodontic* OR "Tooth movement" OR "orthodontic tooth movement" OR "Tooth displacement " OR "orthodontic Treatment" OR "orthodontic Therapy" #2 accelerat* OR rapid* OR short* OR speed* OR fast OR velocity OR duration OR rate OR time OR "regional accelerated phenomenon" OR RAP. #3 laser OR high intensity laser therapy OR HELT OR hard laser OR high-energy laser OR Erbium lasers OR Er: YAG laser OR Er,Cr:YSGG laser OR CO2 laser OR Nd:YAG laser. #4 laser-assisted corticotomy OR lasersicion OR laser-assisted* OR laser induced* OR laser decortication. #5 #3 OR #4 #6 #1 AND #2 AND #5
Google Scholar	#1(orthodontic OR "Tooth movement") AND (accelerate OR acceleration OR accelerating OR duration OR rate) AND (laser OR high intensity laser therapy OR HELT OR hard laser OR high-energy laser OR Erbium lasers OR Er: YAG laser OR Er,Cr:YSGG laser OR CO2 laser OR Nd:YAG laser) #2 (orthodontic OR "Tooth movement") AND (accelerate OR acceleration OR accelerating OR duration OR rate) AND (laser-assisted corticotomy OR lasersicion OR laser-assisted* OR laser induced* OR laser decortication)
Scopus	#1TITLE-ABS-KEY (orthodontic* OR "Tooth movement" OR "orthodontic tooth movement” OR "Tooth displacement “OR "orthodontic Treatment” OR "orthodontic Therapy"). #2 TITLE-ABS-KEY (accelerat* OR rapid* OR short* OR speed* OR fast OR velocity OR duration OR rate OR time OR "regional accelerated phenomenon" OR RAP) #3TITLE-ABS-KEY (laser OR high intensity laser therapy OR HELT OR hard laser OR high-energy laser OR Erbium lasers OR Er: YAG laser OR Er,Cr:YSGG laser OR CO2 laser OR Nd:YAG laser). #4 TITLE-ABS-KEY (laser-assisted corticotomy OR lasersicion OR laser-assisted* OR laser induced* OR laser decortication). #5 #3 OR #4 #6 #1 AND #2 AND #5
Web of Science	#1TS= (orthodontic OR "Tooth movement" OR "orthodontic tooth movement” OR "Tooth displacement “OR "orthodontic Treatment" OR "orthodontic Therapy"). #2TS= (accelerat* OR rapid* OR short* OR speed* OR fast OR velocity OR duration OR rate OR time OR "regional accelerated phenomenon" OR RAP). #3TS= (laser OR high intensity laser therapy OR HELT OR hard laser OR high-energy laser OR Erbium lasers OR Er: YAG laser OR Er,Cr:YSGG laser OR CO2 laser OR Nd:YAG laser). #4 TS= (laser-assisted corticotomy OR lasersicion OR laser-assisted* OR laser induced* OR laser decortication). #5 #3 OR #4 #6 #1 AND #2 AND #6
PQDT OPEN	#1(orthodontic OR "Tooth movement") AND (accelerate OR acceleration OR accelerating OR accelerated OR rapid OR speed OR fast OR quick OR velocity OR duration OR rate OR time OR "regional accelerated phenomenon" OR RAP) AND (laser OR high-intensity laser therapy OR HELT OR hard laser OR high-energy OR laser OR Erbium lasers OR Er: YAG laser OR Er,Cr:YSGG laser OR CO2 laser OR Nd:YAG laser) #2 (orthodontic OR "Tooth movement") AND (accelerate OR acceleration OR accelerating OR accelerated OR rapid OR speed OR fast OR quick OR velocity OR duration OR rate OR time OR "regional accelerated phenomenon" OR RAP) AND (laser-assisted corticotomy OR lasersicion OR laser-assisted* OR laser induced* OR laser decortication)
Trip	(orthodontic OR "Tooth movement") AND (accelerate OR acceleration OR accelerating OR accelerated OR rapid OR speed OR fast OR quick OR velocity OR duration OR rate OR time OR "regional accelerated phenomenon" OR RAP) AND (laser OR high-intensity laser therapy OR HELT OR hard laser OR high-energy OR laser OR Erbium lasers OR Er: YAG laser OR Er,Cr:YSGG laser OR CO2 laser OR Nd:YAG laser OR laser-assisted corticotomy OR lasersicion OR laser-assisted* OR laser induced* OR laser decortication)

Study Selection and Data Extraction

Two reviewers (RIS and MYH) independently assessed the articles for suitability according to the selection criteria; a third reviewer (GM) was asked to decide in case of disagreement. Initially, the titles and abstracts of articles were checked by the two reviewers during the search by using the eligibility criteria. Then, the same two reviewers evaluated the full text of all articles that might be included in the review or if the title or summary was ambiguous to reach a clear judgment. If any article did not fulfill one or more of the eligibility criteria, it was discarded from the review. Finally, the same two authors (RIS and MYH) conducted data extraction in the piloted and predefined data extraction tables. The data extraction sheet contained the following elements: general information (author's name, publication year, and place of study); method (study design, comparison group); participants (sample size (Male/Female), mean age, malocclusion); interventions (type of laser, type, and site of intervention); outcomes (primary and secondary outcomes); and results (the main finding). Other information about orthodontic aspects (appliance characteristics and biomechanics, anchorage tools, frequency of orthodontic adjustments, follow-up time, methods of outcome measurements) was also included.

Assessing the Risk of Bias of the Included Studies

The two reviewers (RIS and MYH) independently assessed the risk of bias of all included studies using Cochrane’s risk of bias tool for randomized trials (RoB2) and the risk of bias in non-randomized studies of interventions (ROBINS-I) tool for non-randomized controlled trials [21-22]. Then, the judgments of both reviewers were compared, a third reviewer (GM) was asked to decide in case of disagreement and could not reach a consensus by discussing. For randomized trials, the following fields were judged as having a high, low, or unclear risk of bias: Randomization process, Deviations from intended interventions, missing outcome data, Measurement of the outcome, Selection of the reported result. Then, the overall risk of bias for each trial was reported according to the following criteria: low risk of bias was reported if all fields were assessed as having a low risk of bias; moderate risk of bias was reported if one or more fields were assessed as having an unclear risk of bias; high risk of bias was reported if one or more fields were assessed as being at high risk of bias.

Results

Literature Search Flow and the Retrieved Studies

**Figure 1:**
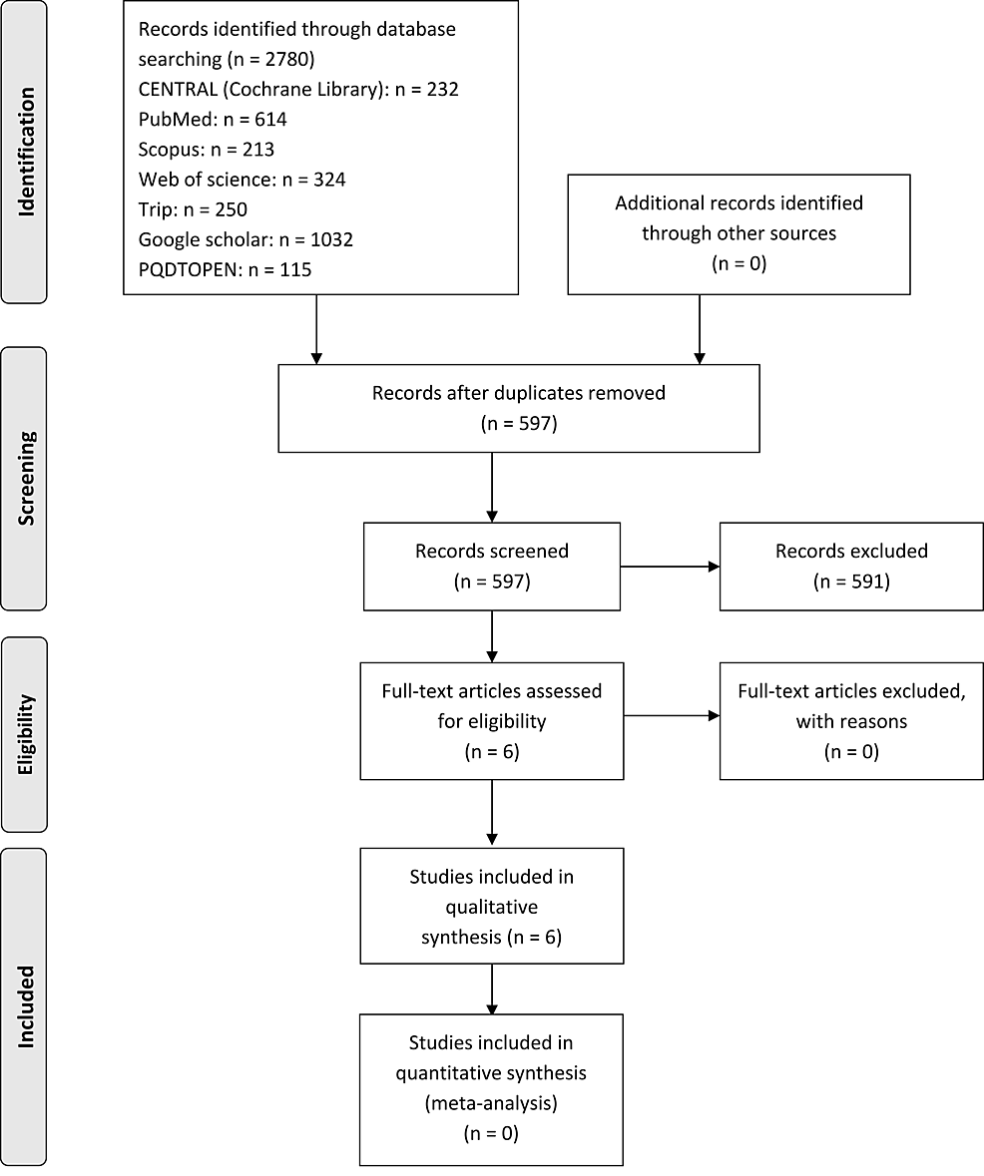
PRISMA 2009 flow diagram of the included studies PRISMA: Preferred Reporting Items for Systematic Reviews

A total of 2780 references were found in the electronic search. Five hundred ninety-seven citations were carefully checked after duplicate references were removed. The titles and abstracts were checked for eligibility and all papers that did not meet the selection criteria were discarded. As a result, six studies were left for full-text assessment. No studies were excluded. Finally, six studies were included in the systematic review [13,16,18,23-25]. Figure 1 shows the Preferred Reporting Items for Systematic Reviews (PRISMA) flow diagram.

Characteristics of the Included Studies

An overview of the characteristics of the six included studies is given in Table 2. Out of the six included trials, five were RCTs. However, the designs of these studies were not similar. One RCT was a parallel-group design with a control group of non-accelerated tooth movement [18], two RCTs were compound trials (two-arm parallel-group design with a split-mouth design for each arm [13,23], and the last two RCTs had a split-mouth design [24-25]. In addition, the only CCT in this systematic review also had a split-mouth design [16]. All of these studies were written in English. All of the studies included males and females without any gender preference. Patient ages ranged between 16.9±2.5 and 21.7 years, noting that the study of Al-Jundi et al. did not mention the mean age of its patients [18].

**Table 2:** Characteristics of included studies in the systematic review RCT: randomized controlled trial, CCT: clinical controlled trial, OT: orthodontic treatment, (M/F): male/female, Exp: experimental group, S.S: stainless steel, NR: not reported

Study/ setting		Methods		Participants	Interventions					
Study	Country	Study design	Treatment comparison	Patients (M/F) Mean age (years) Malocclusion	Type of laser	Type and site of intervention	Appliance characteristics	Anchorage	Orthodontic adjustments	Follow-up
Jaber et al. 2021 [24]	Syria	RCT split-mouth	Er: YAG laser + OT vs. OT	Patients (M/F): 18 (7/11) Control: 18, Exp: 18 Mean age: 16.9 ± 2.5 Malocclusion: class II division 1	Er: YAG (2.94 µm) with two hand-pieces (2060): 200 mJ, 12 Hz (2062): 100 mJ, 10 Hz	8 small perforations (3-mm depth) in the buccal gingiva, 4 at the first premolar extractions sites and the other four were around the canine.	- 0.022-inch slot MBT brackets - Elastic chains from canine brackets to first molars bands, with 150-g force for retraction.	Self-drilling miniscrew (1.3 * 8 mm) between the maxillary 2nd premolar and the first molar.	every 2 weeks	Till the completion of space closure
Mahmoudzadeh et al. 2020 [25]	Iran	RCT split-mouth	Er: YAG laser + OT vs. OT	Patients (M/F): 12(3/9) Control: 12, Exp: 12 Mean age: 18.91±3.87 Malocclusion: Patients scheduled for bilateral extraction of premolars followed by canine retraction	Er, Cr: YSGG laser 2780 nm 3.5 W, 30 Hz, 40% air, 80% water using the MZ5 tip with 500 μ diameter.	A vertical incision (2 to 3 mm depth) in the buccal gingiva parallel to the mesial and distal surfaces of canine root 1 mm below the alveolar crest to the mucogingival junction.	-0.022-inch slot MBT brackets 0.016 × 0.022 S.S wire inserted After the leveling and alignment, a nickel-titanium closed-coil spring (150 g force to each side) was used for canine retraction.	NR	After one month only	One month
Alfawal et al. 2020 [23]	Syria	Compound study	Piezocision + OT vs. laser-assisted flapless corticotomy + OT vs. OT	Patients (M/F): 32 (13/19) Piezocision: 16, LAFC: 16 Mean age: 18.25± 3.5 Malocclusion: class II division 1	Er: YAG laser with R14C handpiece two parameters were used: 100 mJ, 10 Hz, 2 W, then 200 mJ, 12 Hz, 3 W	Piezocision (upper canines); 2 corticotomies (3-mm depth and 10-mm length) in the buccal at equal distance from the upper canine and second premolar. -LAFC (upper canines); 5 small perforations (3-mm depth; 1.3-mm Wide and away from others 1.5- 2 mm) at equal distance from the upper canine and second premolar.	-0.022-inch slot MBT brackets - a nickel-titanium closed-coil spring (150 g force to each side) was used for canine retraction	Soldered transpalatal arches	every 2 weeks	Till the completion of space closure
Al-Jundi et al. 2018 [18]	Syria	RCT	Er: YAG laser + OT vs. OT	Patients (M/F): 30 Control: 15, Exp: 15 Mean age: NR Malocclusion: Deep bite	Er:YAG (2.94 µm) with two hand-pieces (2060) (2062) (2062): 400 mJ, 10 Hz in a pulsed mode, 5 seconds, 4W, pulse duration: 300 µs, Power density: 100 W/cm^2^ and fluence: 10 J/cm^2^ (2060): 400 mJ, 15 Hz, 5 seconds, average power 6W, pulse duration: 300 µs, Power density: 150 W/cm^2^ and fluence: 10 J/cm^2^	Perforations in the cortical bone using a 2060 handpiece on the buccal side according to the vertical imaginary guiding lines, which were parallel to the long axis of the upper incisors' roots and by the horizontal parallel lines, which formed multiple 3 × 4 mm rectangles.	0.022-inch slot MBT brackets - After the leveling and alignment, the upper incisors intrusion commenced using an intrusion 0.016 0.022 S.S wire with T loops and a constant force of 50 g	self-drilling mini-implants between the upper central and lateral incisors	every 3 weeks	Till the completion of intrusion of the upper incisors
Alfawal et al. 2018 [13]	Syria	Compound study	Piezocision + OT vs. laser-assisted flapless corticotomy + OT vs. OT	Patients (M/F): 36 (24/12) Piezocision: 18, LAFC: 18 Mean age: 18.08 ± 3.5 Malocclusion: class II division 1	Er: YAG laser with R14C handpiece, two parameters were used: 100 mJ, 10 Hz, 2 W, then 200 mJ, 12 Hz, 3 W	Piezocision (upper canines); 2 corticotomies (3-mm depth and 10-mm length) in the buccal at equal distance from the upper canine and second premolar. -LAFC (upper canines); 5 small perforations (3-mm depth; 1.3-mm Wide and away from others 1.5- 2 mm) at equal distance from the upper canine and second premolar.	-0.022-inch slot MBT brackets - a nickel-titanium closed-coil spring (150 g force to each side) was used for canine retraction	Soldered transpalatal arches	every 2 weeks	Till the completion of space closure
Salman and Ali 2014 [16]	Iraq	CCT, split-mouth	Laser-assisted corticotomy + OT vs. OT	Patients (M/F): 15 (5/10) Mean age: 21.7 Malocclusion: Class I or Class II malocclusion cases that require bilateral extraction of maxillary premolar	-Soft tissue incision by KAVO laser device using a special handpiece with a fiber-optic delivery system. -Er: YAG laser using parameters for bone ablation and another type of handpiece	4 perforations (3-mm depth; 1.5-mm in diameter and away from others 2-3 mm) between maxillary lateral incisor and canine and the other was between maxillary canine and the 2nd premolar.	NR	NR	NR	Six weeks after surgery

Extraction treatment (maxillary first-premolar extraction followed by a canine retraction) was performed in five studies [13,16,23-25], and only one study evaluated the effect of HELT-based flapless corticotomy on upper incisors' intrusion; it was a non-extraction-based study [18].

A pre-adjusted orthodontic appliance (MBT 0.022 × 0.028-inch slots) was used in the five trials to investigate the effects of HELT on tooth movement [13,18,23-25]. The sixth study by Salman and Ali did not report any information about the type and prescription of brackets [16]. The canine retraction was done using a nickel-titanium closed-coil spring, which applied 150 g force, in three studies [13,23,25]. Jaber et al. used elastic chains that extended from canine brackets to the first molars bands, with 150 g force to retract canines [24]. The upper incisors intrusion was commenced using 0.016 × 0.022-inch stainless steel archwire with T-loops, which applied a constant force of 50 grams on each side in the Al-Jundi et al. trial [18]. Of note, the study by Salman and Ali was lacking this information [16].

Two types of erbium laser were evaluated (erbium-doped yttrium aluminum garnet (Er: YAG) [13,16,18,23-24] and erbium, chromium-doped yttrium scandium gallium garnet (Er, Cr: YSGG) [25]. The Er: YAG laser (2.94 µm) was used in five studies. The KAVO laser device (Biberach an der Riss, Germany) with two hand-pieces (2062 and 2060) was used in three studies [16,18,24], whereas the LightWalker®ST-E Fotona device (Dallas, Texas) with an R14C hand-piece was used by Alfawal et al. [13,23].

Different laser parameters were also used by researchers; Al-Jundi et al. used 400 mJ/10 Hz/4W for gingival perforations, then 400 mJ/15 Hz/6W for alveolar bone perforations [18]. However, Alfawal et al. used these parameters: 100 mJ/10 Hz/2 W, then 200 mJ/12 Hz/3 W for gingival and alveolar bone penetration, respectively [13,23]. The parameter 200 mJ/10 Hz, followed by the parameter 100 mJ/12 Hz were used by Jaber et al. depending on the handpiece used [24].

On the other hand, Mahmoudzadeh et al. used Er, Cr: YSGG laser to accelerate canine retraction movement. In this study, the Waterlase iPlus Biolase device (Foothill Ranch, California) was used with the following parameters: 3.5 W, 30 Hz, H’ mode 40% air and 80% water, using the MZ5 tip (500 μ diameter) [25].

The primary outcome was the rate of canine movement in three studies [13,24-25], the net canine movement in one study [16], and the time of teeth movement in one study [18]. However, the primary outcome in the sixth study of Alfawal et al. [23] was a secondary outcome in the current review (i.e., patient-centered outcomes associated with upper canine retraction). The secondary outcome, such as pain, was evaluated in four studies [18,23-25]. Anchorage loss and undesirable tooth movements were evaluated in two studies [13,25], and periodontal problems were evaluated in two studies [16,25].

The follow-up lasted until the completion of the intended tooth movement in four studies and ranged from two to four months [13,18,23-24], whereas Mohmoudzadeh M et al. followed up the patients for one month only [25]. Finally, the trial of Salman and Ali followed the recruited patients for only six weeks [16]. The included studies were published from 2014 to 2021.

Risk of Bias in the Included Studies

**Figure 2:**
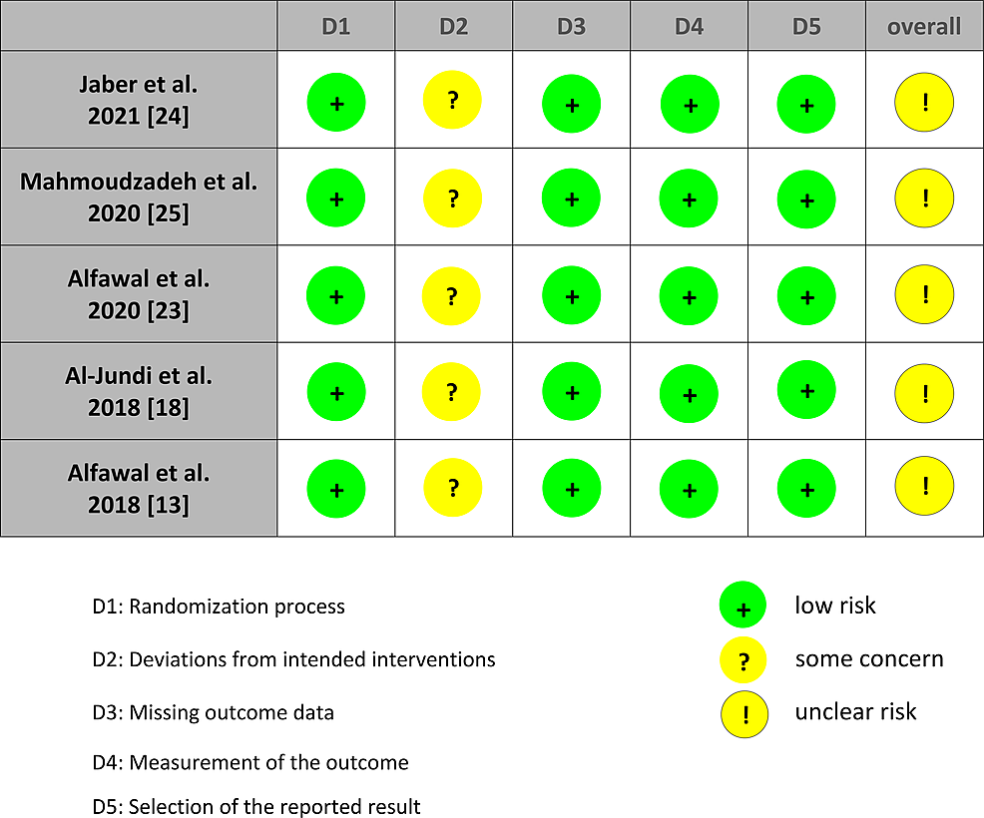
Risk of bias summary: the review authors’ judgments about each item of the risk of bias for the included studies

**Figure 3:**
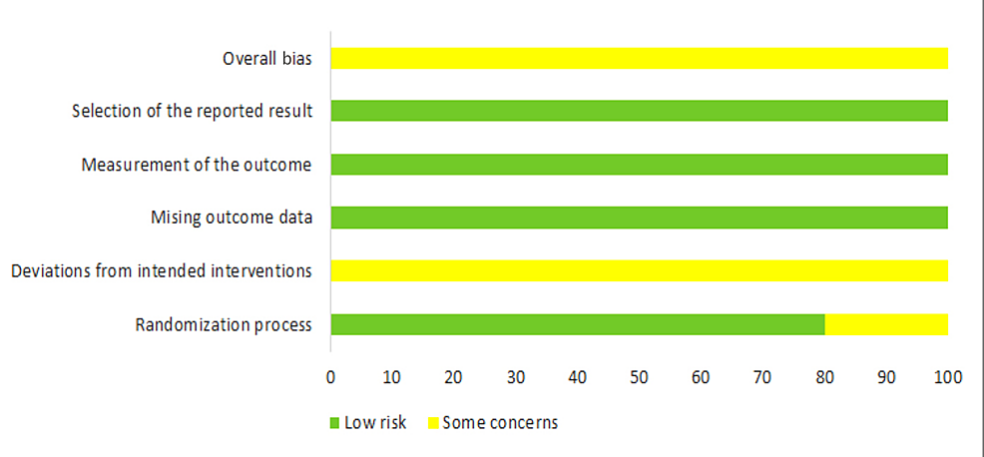
Risk of bias graph: the review authors’ judgments about each item of the risk of bias, presented as percentages across all the studies included

The summary of the overall risk of bias of the included RCTs is shown in Figures 2-3. The five RCTs were at unclear risk of bias, whereas the only CCT was also at moderate risk of bias. The five included RCTs adequately addressed sequence generation (randomization). For blinding, only the outcome assessor's blinding was possible in these studies because the included patients and the investigators could not be blinded regarding the surgical intervention performed. For missing data and selective reporting bias, all studies were at low risk of bias. More information about the risk of bias assessment, along with the reasons supporting each assessment can be found in Appendices 1 and 2.

Effects of Interventions

Primary outcome: Rate of tooth movement. Out of the six included trials, four trials assessed the effect of erbium lasers on canine retraction speed after premolar extraction [13,16,24-25], the fifth trial evaluated the effect of Er: YAG laser on the incisors intrusion rate in a non-extraction treatment [18]; Table 3 and Appendix 3.

**Table 3:** Results of the included studies in this systematic review CRR: canine retraction rate, CMR: canine movement rate, TTM: time of teeth movements, NCM: net canine movement, MAL: molar anchorage loss, CR: canine rotation

Study/setting		Outcomes			Results	
Study	Country	Primary outcomes	Secondary outcomes	Methods of outcomes measurements	Primary outcomes	Secondary outcomes
Jaber et al 2021 [24]	Syria	Canine retraction rate	Levels of pain and discomfort during the first week after laser application	Primary outcome: Digital Boely gauge: immediately after laser application, one, two, four, eight, and 12 weeks. Secondary outcome: A questionnaire on the 1st, 3rd, 5th, and 7th days after laser application.	CRR: Significant differences were observed (P< 0.001) at the 1^st^ and 2nd months. No significant difference was found at the 8th- to 12th-week interval.	Levels of pain and discomfort: A significant reduction was seen in the mean score of pain during eating at all assessment times when compared to the baseline data (P=0.002 at day 2, P<0.001 at days 5 and 7).
Mahmoudzadeh et al 2020 [25]	Iran	Canine movement rate	Canine rotation, the rate of anchorage control, the level of pain, and the GI.	Primary outcome: distance between the cusp tip of the canine and the rugae line in scanned casts. Secondary outcome: scanned casts (canine rotation, anchorage control), modified McGill pain questionnaire.	CMR: Significant differences were observed (P< 0.001) in the 1st month.	MAL: insignificant differences were observed (P= 0.68) at the 1st month. CR: Significant differences were observed (P= 0.029) at the 1st month in the experimental sides. Levels of pain: only one patient reported pain.
Alfawal et al 2020 [23]	Syria	Patient-centered outcomes associated with canine retraction accelerated by using piezocision or LAFC.		Primary outcome: Standardized questionnaires using the numerical rating scale (NRS) at four time points: 24 h (T1); 3 days (T2); 7 days (T3); and 14 days (T4).	The levels of pain, discomfort, swelling, and difficulty in chewing were significantly greater at the experimental side only at T1 in both groups (p < 0.05).	
Al-Jundi et al 2018 [18]	Syria	Time of teeth movements.	Pain perception and satisfaction	Primary outcome: lateral cephalometric radiographs {Before treatment (T1), after finishing the leveling and alignment (T2), and after completion of the intrusion (T3).} Secondary outcome: a visual pain scale {on day 1(T1), on day 3 (T2), and on day 7 (T3).}	TTM: There was a significant positive difference in the experimental group. The mean increase was 38.4%, approximately 3 times faster.	Level of pain: was significantly lower in the experimental group on Days 3 and 7.
Alfawal et al 2018 [13]	Syria	Rate of canine movement.	Molar anchorage loss, canines’ rotation, and the duration of canine retraction.	Primary outcome: distance between the cusp tip of the canine and the rugae line in photographed casts. Secondary outcome: photographed casts (canine rotation, anchorage control). Model casts were taken 1 month (T1), 2 months (T2), 3 months (T3), and 4 months (T4) following the onset of canine retraction.	CMR: were significantly higher in the experimental sides during the first 2 months in both groups (p < 0.001).	CR: were greater in the experimental sides, however, these differences were insignificant (p > 0.05) MAL: there were no significant differences(p > 0.05) * No harms were observed
Salman & Ali 2014 [16]	Iraq	Net of canine movement.	Pulp vitality, gingival health, and pocket depth	-Periapical radiography - vitality testing - gingival sulcus depth - model casts	NCM: Higher mean value of retraction has shown on the laser corticotomy side.	Pulp vitality response and post-surgery gingival sulcus depth showed no significant difference between the pre-laser and post-laser surgery.

The effect of erbium lasers in accelerating upper canine retraction: three split-mouth trials [16,24-25] and one compound trial [13] assessed the efficacy of erbium lasers in accelerating upper canine retraction. Noteworthy, the results could not pool in a meta-analysis because of the differences in the way of orthodontic force delivery, types and parameters of lasers, and laser application protocol.

The amount of maxillary canine retraction at the first month was assessed by 3 trials [13,24-25], comprising 94 left and right canines. There was a greater canine retraction (1.57±0.36, 1.21±0.35, 1.95±0.22 mm, respectively) in the laser-assisted flapless corticotomy group (p<0.001) compared to the control group. According to the GRADE (Grading of Recommendations, Assessment, Development, and Evaluations) approach, the quality of evidence supporting this outcome was moderate (Table 4).

**Table 4:** Summary of findings according to GRADE guidelines C: confidence interval; PGD: parallel-group design; SMD: split-mouth design a Decline one level for risk of bias (unclear risk of bias of deviations from intended intervention in [13,24-25]) and one level for indirectness* b Decline one level for risk of bias (unclear risk of bias of deviations from intended intervention in [13,24]), one level for indirectness*, and one level for imprecision** c Decline one level for risk of bias (unclear risk of bias of deviations from intended intervention in [18]), one level for indirectness*, and one level for imprecision**. d Decline one level for risk of bias (unclear risk of bias of deviations from intended intervention in [18,23-25]), one level for indirectness*, and one level for imprecision**. e Decline one level for risk of bias (unclear risk of bias of deviations from intended intervention in [13,25]), one level for indirectness*, and one level for imprecision**. f Decline two levels for risk of bias (moderate risk of bias in classification of interventions [16], unclear risk of bias of deviations from intended intervention [25]), one level for imprecision**, and one level for indirectness*. *Outcome is not directly related; the included trials involved only adult patients, so the efficacy of Er: YAG radiation could not be confirmed on adolescent patients. Also, patient-centered outcomes were very limited. **Limited number of trials, of limited sample size GRADE: Grading of Recommendations, Assessment, Development, and Evaluations

No. of studies	No. of patients	Weighted mean difference (95% CI)	Quality of the evidence (GRADE)	Comments
Upper canine retraction facilitated by Er: YAG laser (month 1)				
3 RCT	37 patients SMD	Relative effect (95% CI): not estimable	⊕⊕⊝⊝ Moderate ^a^	There was a significant difference between the conventional and experimental groups.
upper canine retraction facilitated by Er: YAG laser (month 2)				
2 RCT	35 patients SMD	Relative effect (95 % CI): not estimable	⊕⊝⊝⊝ Low ^b^	Also, this outcome was assessed at 3 months in 2 studies (35 patients). the difference was not significant between both groups (−0.11 lower to 0.12 higher) with a quality of evidence low ⊕⊝⊝⊝^b^.
Upper incisors intrusion facilitated by Er: YAG laser				
1 RCT	30 patients PGD	Relative effect (95 % CI): not estimable	⊕⊝⊝⊝ low ^c^	There was a significant difference in the treatment time between the conventional and experimental groups (95.8 vs. 59 days, respectively, the time for the treatment in the experimental group was 38.4% less compared with the control group).
Pain and discomfort:				
4 RCT	76 patients (3 RCTs SMD, and 1RCT PGD )	Relative effect (95 % CI): not estimable	⊕⊝⊝⊝ low ^d^	The levels of experienced pain and discomfort were significantly greater at the experimental sides as compared to the control sides on the first day only (p = 0.005 and p < 0.001, respectively). We could not pool the results of the previous 4 trials which evaluated this outcome to quantitative synthesis due to differences in specific treatments (non-extraction vs. extraction) and evaluation tools.
Anchorage loss				
2 RCT	29 patients SMD	Relative effect (95 % CI): not estimable	⊕⊝⊝⊝ low ^e^	There were no significant differences between the experimental and control sides during the four evaluation times (p > 0.05).
undesirable tooth movements (canines’ rotation)				
2 RCT	29 patients SMD	Relative effect (95 % CI): not estimable	⊕⊝⊝⊝ low ^e^	The differences between the experimental and control sides were negligible and insignificant (p > 0.05).
Periodontal problems				
1 RCT and 1 CCT	27 patients SMD	Relative effect (95 % CI): not estimable	⊝⊝⊝⊝ very low ^f^	There is no significant change in the gingival sulcus depth, width of attached gingiva, and gingival index pre and post-surgery.

The study by Salman and Ali also found that the canine in the laser side moved twice the amount of that in the control side after six weeks of observation [16]. Two trials assessed the cumulative maxillary canine movement following the first-premolar extraction with two months observation period [13,24], comprising 70 left and right canines. There was a greater canine retraction (1.25±0.30, 0.40±0.18 mm, respectively) in the laser-assisted flapless corticotomy group (p< 0.001). According to GRADE, the quality of evidence supporting this outcome was low (Table 4).

The degree of retraction at the third month was assessed by two trials [13,24], comprising 70 canines. The retraction rate on the experimental side was almost equal to its rate on the control side (p=0.220, p=0.427, respectively). According to GRADE recommendations, the strength of evidence supporting this outcome was low (Table 4).

Effect of Er: YAG laser in accelerating upper incisors intrusion. One trial conducted by Al-Jundi et al. evaluated the acceleratory effect of using the Er: YAG laser in flapless corticotomy at the anterior regions of the maxilla for incisor intrusion [18]. This study was performed on 30 adult patients with a deep overbite. An intrusion arch with T loops of 0.016*0.022 stainless steel wire and attached to mini-implants of self-drilling type inserted between the upper central and lateral incisors by an elastic chain was used to accomplish incisor intrusion in patients. The Er: YAG laser was started on the same day as the placement of the intrusion arch. This study reported a significant difference in treatment time between the conventional and experimental groups (3.78 mm in 95.8 days vs. 4.587 mm in 59 days, respectively). The time for the treatment in the experimental group was 38.4% less compared with the control group.

Secondary Outcomes

Pain and discomfort:** **Four trials evaluated the levels of pain and discomfort associated with erbium laser radiation [18,23-25]. However, the results could not be pooled to quantitative synthesis due to the use of different scales for pain assessment.

Alfawal et al. [23] evaluated pain and discomfort levels associated with laser-assisted flapless corticotomy during canine retraction using self-reported questionnaires. The levels of pain and discomfort were significantly higher in the experimental group on the first day compared to the control group (mean NRS difference was 4 and 5.5 with P-values of 0.005 and <0.001 for pain and discomfort, respectively). Then, the levels of experienced pain and discomfort dramatically decreased in the experimental group at three days (T2); seven days (T3); and 14 days (T4), and insignificant differences were reported between the two sides.

A five-item questionnaire was used by Jaber et al. [24] to assess the levels of pain and discomfort during canine retraction. They found that 11.1% and 44% of the patients experienced severe and mild pain during the first day, respectively. On the third day, 72.2% of the patients were free of pain, and this percentage rose to 83.3% on the seventh day. Of the 12 patients examined by Mahmoudzadeh et al. [25], only one patient reported mild pain in the laser side after the wire insertion (visual analog score (VAS) score: 2), and it lasted for less than one day. Al-Jundi et al. assessed pain during acceleration of tooth intrusion [18]. There was no significant increase in pain scores. On Day 3 as on Day 7, the pain score in the experimental group was significantly lower as compared with the control group. According to GRADE recommendations, the strength of the evidence supporting this outcome was low (Table 4).

Anchorage loss and undesirable tooth movements: Only two trials evaluated these variables [13,25]. Alfawal et al. and Mahmoudzadeh et al. investigated molar anchorage loss and upper canines' rotation during their retraction (Table 3).

For molar anchorage loss, the differences between the experimental and control sides were not significant during the observation time (p>0.05 and p= 0.680, respectively). The observation time in the study of Mahmoudzadeh et al. [25] was only one month. According to GRADE, the evidence supporting this outcome is very low (Table 4).

The rates of canines’ rotation were greater in the experimental sides compared to the control sides in both trials. However, these differences were insignificant (p>0.05) after four months of observation in the Alfawal et al. trial [13]. There was very low evidence supporting this outcome according to GRADE (Table 4).

Periodontal problems: These problems were evaluated in two studies [16,25]. Salman and Ali compared mean gingival sulcus depth values of retracted canines pre and post-surgery and found clinically insignificant differences (i.e. less than 4 mm) [16]. The width of the attached gingiva and gingival index were evaluated by Mahmoudzadeh et al. who did not find any significant difference between the laser and control sides in these measures [25].

Discussion

To our knowledge, this is the first systematic review in the literature evaluating the effectiveness of high-energy lasers therapy (HELT) with flapless corticotomy in accelerating orthodontic tooth movement. The present review performed an overall qualitative assessment of the currently available studies, which comprised 155 patients from six trials. In order to minimize bias and possible confounders, only randomized and non-randomized controlled trials were included. The six trials that evaluated HELT were judged to be at unclear risk of bias; Participant blinding was the most problematic field. So this has affected the level of certainty of the achieved results.

Four trials investigated the efficacy of erbium lasers irradiation in accelerating canine retraction after premolar extraction [13,24-25]. They reported greater tooth movement with the erbium laser as compared to the conventional method by 2- and 1.5-fold in the first and second months, respectively. However, the retraction rate on the experimental side was almost equal to its rate on the control side at the third month of intervention. This acceleration can be explained by the RAP phenomenon that was induced by laser-assisted corticotomy and decreased resistance of the alveolar bone to tooth movement [17,26]. In addition, the increased expression of inflammatory markers and cytokine levels stimulated by the selective removal of alveolar bone may have led to an increase in osteoclast activity, which, in turn, may have enhanced bone remodeling and accelerated tooth movement [27-28]. The temporary nature of the RAP could also explain why the acceleration occurred in the first two months only and then the canine retraction speed decreased gradually.

With erbium lasers, the peak of the RAP was after a month and decreased at the end of the second month. However, Wilcko et al. reported that the RAP phenomenon starts within a few days following injury, reaches its peak after four to eight weeks, and lasts for two to four months [26,29]. This difference with laser-assisted flapless corticotomy could be attributed to the less aggressive nature of this intervention compared to that of Wilcko. On the other hand, the present findings corroborate the results of Alfawal et al. who reported a significant reduction in treatment time when using minimally invasive flapless techniques for corticotomy like micro-osteoperforations or piezocision during teeth movement [6]. They showed that the tooth movement increased in the first two months, which is similar to what was found in this systematic review.

Al-Jundi et al. evaluated the effectiveness of flapless corticotomy with Er: YAG laser in accelerating incisors intrusion (non-extraction treatment) and reported a significant difference in the overall treatment time; the mean increase in the rate of tooth movement was 38.4% (95.8 and 59 days, respectively) [18]. When comparing the findings of this non-extraction-based trial (the tooth movement was approximately 2.5 times faster in the experimental group) [18] with the three previous extraction-based trials (the tooth movement was approximately two times faster in the experimental group) [13,24-25], it seems that the types of movements did not influence the accelerating rate by the erbium laser.

One hundred fifty-five participants were included in the six studies. The six trials included only adult patients. The previously included trials evaluated a variety of flapless corticotomy protocols with variations in the design and size of the cortical bone cuts. Therefore, future studies must test the effect of these differences on the amount of acceleration and the adverse side effects of each individual intervention.

The levels of pain and discomfort associated with HELT were higher at the experimental sides than those at the control sides on the first day only [23-24]. The trauma of the alveolar bone and gingiva after surgery and the associated increase in inflammatory markers and cytokine levels can explain these slightly greater levels of perceived pain during the application of laser. When comparing the levels of pain and discomfort associated with laser-assisted flapless corticotomy with conventional corticotomy [4], no significant levels of pain and discomfort associated with erbium laser radiation were reported. This can be explained by the conservative and less invasive nature of this irradiation. Also, no flap reflection or sutures were required. Another assumption is related to the sensory nerve endings that may have been blocked by the protein coagulation caused by laser cutting and thus relieving the sensation of pain [30]. Furthermore, the non-contact mode used with laser-assisted flapless corticotomy was accompanied by no mechanical pressure on the gingival tissue as opposed to the traditional corticotomy, which resulted in less discomfort.

Although no important levels of pain and discomfort associated with laser-assisted flapless corticotomy were reported in the evaluated trials, the evidence is weak. Therefore, further trials should investigate this outcome as well as other patient-reported measures.

Only two included trials investigated the undesirable tooth movements (canine rotation, molar anchorage loss) associated with laser-assisted flapless corticotomy [13,25]. Insignificant differences between the experimental and control groups were reported about the loss of anchorage. The anchorage loss rate ranged from 0.11 to 0.61 mm/month. However, these mean values are considered clinically insignificant and can be attributed to the conservative and less invasive nature of laser-assisted flapless corticotomy. Minimal weakening of the alveolar cortical bone may have allowed the upper canines to retract without exerting enough forces to allow mesial drifting of the posterior anchoring teeth.

Higher canines’ rotation rate was reported on the surgical side compared to the control side [13,25]. However, this increase was not significant and could be negligible. This may be attributed to the high retraction rate and least alveolar bone density on the surgical site so that the movement of the teeth became easier and the surrounding structures were less resistant.

The included trials did not report any adverse effects of laser-assisted flapless corticotomy on the periodontal tissue [25]. Salman and Ali evaluated only the gingival sulcus depth to assess periodontal changes following the acceleratory intervention [16], whereas the width of the attached gingiva and the gingival index were evaluated by Mahmoudzadeh et al. [25]. However, they did not assess other important variables such as plaque index, bleeding index, and gingival recession. Therefore, future research work should place more emphasis on the possible side effects of laser-assisted flapless corticotomy on periodontal tissues.

Limitations

A lack of large, high-quality studies investigating HELT-based flapless corticotomy in the acceleration of tooth movement is evident. Altogether, most of the included studies were at unclear risk of bias and had small sample sizes. Most studies evaluated part of the provided orthodontic treatment and not the entire treatment duration. Adverse effects are investigated in a limited number of studies, and there have been no attempts to assess the interventions in terms of cost-benefit analysis. Long-term follow-up of the response to these interventions was also lacking among the included studies.

## Conclusions

The efficacy of HELT with flapless corticotomy in accelerating tooth movement appeared to be significant at least in the first two months according to this review. However, the evidence has been found low to moderate according to the GRADE approach. More well-conducted studies are needed, with more attention paid to the size of the sample, the overall follow-up period, the surgical protocol (site, size, and design of surgical procedure, and the number of surgical interventions), the type of orthodontic treatment (extraction vs. non-extraction), side effects, and cost-benefit ratios.

## Appendices

Appendix 1

**Table 5:** Assessment of the risk of bias with supporting reasons for each assessment

Study	country	D1	D2	D3	D4	D5	Overall bias
Jaber et al. 2021 [24]	Syria	Low risk: Randomization sequences were generated using computer-generated random numbers with an allocation ratio of 1:1. no mention of the method used to conceal the allocation sequence	Some concerns: Blinding of participants and people delivering the intervention cannot be performed.	Low risk: No dropouts were reported	Low risk: “The investigators performing the measurements and data analysis were blinded from the group assignments.” It was possibly done.	Low risk: The protocol for the study was registered in clinical trial.gov study ID: (NCT04316403) and the outcomes mentioned in the protocol have been reported	Some concerns: The study is judged to raise some concerns because one domain got this result
Mahmoudzadeh et al. 2020 [25]	Iran	Low risk: The allocation of patients to the treatment blocks (In the first block, the right quadrant was considered as the control side while the left quadrant was considered as the laser side. In the second block, the left quadrant served as the control side, and the right quadrant was considered as the laser side.) was performed by flipping a coin	Some concerns: Blinding of participants and people delivering the intervention cannot be performed.	Low risk: No dropouts were reported	Low risk: The investigators performing the measurements and data analysis were blinded from the group assignments	Low risk: The protocol for the study was registered in the Iranian Registry of Clinical Trials available at www.irct.ir (identifier: IRCT20120215009014N280)	Some concerns: The study is judged to raise some concerns because one domain got this result
Alfawal et al. 2020 [23]	Syria	Low risk: Randomization sequences were generated using computer-generated random numbers with an allocation ratio of 1:1. The allocation sequence was concealed using sequentially numbered, opaque, sealed envelopes.	Some concerns: Blinding of participants and people delivering the intervention cannot be performed.	Low risk: No patient was lost to follow-up.	Low risk: The investigators performing the measurements and data analysis were blinded from the group assignments	Low risk: The protocol for the study was registered in clinical trial.gov study ID: (NCT02606331) and the outcomes mentioned in the protocol have been reported	Some concerns: The study is judged to raise some concerns in at least one domain for this result.
Al-Jundi et al. 2018 [18]	Syria	Low risk: Randomization sequences were generated using Sealed envelopes containing the random allocation of each patient to one or the other group.	Some concerns: Blinding of participants and people delivering the intervention cannot be performed.	Low risk: No dropouts were reported	Low risk: No details of blinding of outcome assessors. But we judge that the outcome was not likely to be influenced by knowledge of intervention received	Low risk: The protocol was not registered. But the pre-defined outcomes mentioned in the methods section seemed to have been reported.	Some concerns: The study is judged to raise some concerns because one domain got this result
Alfawal et al. 2018 [13]	Syria	Low risk: Randomization sequences were generated using computer-generated random numbers with an allocation ratio of 1:1. The allocation sequence was concealed using sequentially numbered, opaque, sealed envelopes.	Some concerns: Blinding of participants and people delivering the intervention cannot be performed.	Low risk: “2 patients (one patient in each group) were lost to follow up due to personal reasons.” We judge that the outcome is not likely to be influenced.	Low risk: The investigators performing the measurements and data analysis were blinded from the group assignments	Low risk: The protocol for the study was registered in clinical trial.gov study ID: (NCT02606331) and the outcomes mentioned in the protocol have been reported	Some concerns: The study is judged to raise some concerns in at least one domain for this result.

Appendix 2

**Table 6:** Risk of bias of the included CCT in this systematic review CCT: non-randomized controlled trial

Study	Bias due to confounding	Bias in the selection of participants into the study	Bias in the classification of interventions	Bias due to deviations from intended interventions	Bias due to missing data	Bias in measurement of outcomes	Bias in selection of the reported result	Overall
Salman and Ali 2014 [16]	Low No confounding is expected.	Low All participants who would have been eligible for the target trial were included in the study. Furthermore, for each participant, the start of follow-up and the start of intervention coincided.	Moderate Corticotomy was done at the side, having more space between the canine and the second premolar.	Low	Low No dropouts were reported	Low The outcome measure was unlikely to be influenced by knowledge of the intervention received by study participants	Low The protocol was not registered. But the pre-defined outcomes mentioned in the methods section seemed to have been reported.	Moderate

Appendix 3

**Table 7:** A synopsis of quantitative measurements for the primary outcome in each study RCR: rate of canine retraction, CMR: canine movement rate, TTM: time of tooth movement, NCM: net of canine movement, LG: laser group, PG: piezocision group, NR: not reported

Study	country	Primary outcome	Time points of measurement	Surgical group (mean ±SD)		Non-surgical group(mean ±SD)		P-value	
Jaber et al. 2021 [24]	Syria	RCR	0-1 week	0.85 ± 0.21		0.34 ± 0.16		˂0.001	
			1-2 week	0.72 ± 0.20		0.38 ± 0.15		˂0.001	
			2-4 weeks	1.21 ± 0.35		0.69 ±0.34		˂0.001	
			4-8 weeks	0.40 ± 0.18		0.22 ± 0.08		˂0.001	
			8-12 weeks	0.23 ± 0.10		0.26 ± 0.10		˂0.001	
Mahmoudzadeh et al. 2020 [25]	Iran	CMR	Mean of total CMR	9.290 ± 3.49		9.89 ± 2.57		˂0.001	
Alfawal et al. 2020 [23]	Syria	Pain	T1(24 h)	LG Madian/IQR 4/2-5	PG Madian/IQR 5.5/4-7	LG Madian/IQR 2/1.275	PG Madian/IQR 1.5/0.25-2.75	LG 0.005	PG 0.001
			T2(3 days)	1/0-2	1.5/1-2.75	1/0-1	1/0.25-1.75	0.106	0.100
			T3(7 days)	0/0-1	1/0-1	0/0-1	0/0-1	0.157	0.157
			T4(14 days)	0/0-0	0/0-0	0/0-0	0/0-0	0.157	0.317
		Discomfort	T1(24 h)	LG Madian/IQR 5.5/5-7	PG Madian/IQR 8/6-9	LG Madian/IQR 3/2-4	PG Madian/IQR 4/3-5	LG ˂0.001	PG ˂0.001
			T2(3 days)	3/1.25-4	3/2-4.75	2/1-2.75	2/1-3	0.096	0.065
			T3(7 days)	0/0-1.75	0/0-2	0/0-0.75	0/0-1	0.167	0.121
			T4(14 days)	0/0-0.75	0/0-0.75	0/0-0.75	0/0-0	1.000	0.317
Al-Jundi et al. 2018 [18]	Syria	TTM (days)	The mean of total days	59.000 ± 13.496		95.80 ±12.35		NR	
Alfawal et al. 2018 [13]	Syria	RCR (mm/month)	T0-T1 (1st month)	LG 1.57 0.36	PG 1.65 0.40	LG 0.79 0.11	PG 0.83 0.18	LG ˂0.001	PG ˂0.001
			T1-T2 (2nd month)	1.25 0.30	1.38 0.32	0.85 0.14	0.88 0.14	˂0.001	˂0.001
			T2-T3 (3rd month)	1.06 0.28	1.10 0.29	0.96 0.25	0.98 0.22	0.220	0.134
			T3-T4 (4th month)	0.89 0.16	0.87 0.11	0.90 0.16	0.94 0.09	0.791	0.23
			T0-T4	1.14 0.10	1.19 0.16	0.84 0.05	0.90 0.09	0.006	0.007
Salman & Ali 2014 [16]	Iraq	NCM	Mean of total NCM	1.63		0.82		NR	
